# Highly Conductive Fe-Doped (La,Sr)(Ga,Mg)O_3−δ_ Solid-State Membranes for Electrochemical Application

**DOI:** 10.3390/membranes13050502

**Published:** 2023-05-10

**Authors:** Egor Gordeev, Semyon Belyakov, Ekaterina Antonova, Denis Osinkin

**Affiliations:** 1 Laboratory of Electrochemical Devices and Fuel Cells, Institute of High-Temperature Electrochemistry, Ural Branch of the Russian Academy of Sciences, Yekaterinburg 620137, Russia; egorgordeev1998@mail.ru; 2 Scientific Laboratory of Hydrogen Energy, Institute of Hydrogen Energy, Ural Federal University, Yekaterinburg 620002, Russia; 3 Laboratory of Electrochemical Materials Science, Institute of High-Temperature Electrochemistry, Ural Branch of the Russian Academy of Sciences, Yekaterinburg 620137, Russia; bca2@mail.ru; 4 Laboratory of Solid Oxide Fuel Cells, Institute of High-Temperature Electrochemistry, Ural Branch of the Russian Academy of Sciences, Yekaterinburg 620137, Russia; antonova_ek@list.ru; 5 Department of Life Safety, Institute of Fundamental Education, Ural Federal University, Yekaterinburg 620002, Russia; 6 Department of Environmental Economics, Graduate School of Economics and Management, Ural Federal University, Yekaterinburg 620002, Russia

**Keywords:** solid oxide fuel cell, (La,Sr)(Ga,Mg)O_3_, Sr_2_Fe_1.5_Mo_0.5_O_6−δ_, symmetrical electrodes, conductivity, thermal expansion, LSGM, distribution of relaxation times, DRT, oxygen reduction reaction

## Abstract

Membranes based on complex solid oxides with oxygen-ionic conductivity are widely used in high-temperature electrochemical devices such as fuel cells, electrolyzers, sensors, gas purifiers, etc. The performance of these devices depends on the oxygen-ionic conductivity value of the membrane. Highly conductive complex oxides with the overall composition of (La,Sr)(Ga,Mg)O_3_ have regained the attention of researchers in recent years due to the progress in the development of electrochemical devices with symmetrical electrodes. In this research, we studied how the introduction of iron cations into the gallium sublattice in (La,Sr)(Ga,Mg)O_3_ affects the fundamental properties of the oxides and the electrochemical performance of cells based on (La,Sr)(Ga,Fe,Mg)O_3_. It was found that the introduction of iron leads to an increase in the electrical conductivity and thermal expansion in an oxidizing atmosphere, while no such behavior was observed in a wet hydrogen atmosphere. The introduction of iron into a (La,Sr)(Ga,Mg)O_3_ electrolyte leads to an increase in the electrochemical activity of Sr_2_Fe_1.5_Mo_0.5_O_6−δ_ electrodes in contact with the electrolyte. Fuel cell studies have shown that, in the case of a 550 µm-thick Fe-doped (La,Sr)(Ga,Mg)O_3_ supporting electrolyte (Fe content 10 mol.%) and symmetrical Sr_2_Fe_1.5_Mo_0.5_O_6−δ_ electrodes, the cell exhibits a power density of more than 600 mW/cm^2^ at 800 °C.

## 1. Introduction

Simple and complex solid oxides are widely investigated in various fields of science and technology due to their diverse physical and chemical properties [1,2,3]. Complex solid oxides with high oxygen-ionic or protonic conductivity (i.e., electrolytes) are used in electrochemical devices for generating electricity (fuel cells) [4,5], producing gases (electrolyzers) [6,7], their purification (separating membranes) [8,9], and gas phase analysis (sensors) [10,11]. The efficiency of all of these devices depends on the properties of the electrolyte, in particular, on its ionic conductivity. 

Doped complex oxides based on lanthanum gallate (LaGaO_3_) have attracted the attention of researchers as promising electrolytes with high oxygen-ionic conductivity in the temperature range of 500–800 °C [12,13]. Many researchers agree that compositions where 10–20 % of the lanthanum is replaced with strontium, and 15–20% of the gallium is replaced with magnesium ((La,Sr)(Ga,Mg)O_3_–LSGM), have the highest ionic conductivity [14,15]. In recent years, solid electrolytes based on doped lanthanum gallate have received a new round of investigation due to the success in the development of solid oxide fuel cells with symmetrical electrodes, where LSGM is applied as a supporting electrolyte [16,17].

The oxygen-ionic conductivity of LSGM is quite high—several times higher than the ionic conductivity of the conventional Y-doped ZrO_2_ electrolyte—and is comparable with that of the CeO_2_-based (Ce,Sm)O_2−δ_ and Ba(Zr,Ce,Y,Yb)O_3−δ_ complex oxides [18,19]. However, unlike (Ce,Sm)O_2−δ_ and Ba(Zr,Ce,Y,Yb)O_3−δ_, which are ionic conductors in oxidizing and reducing atmospheres, respectively, LSGM is an oxygen-ionic conductor in the wide range of oxygen partial pressures [20,21]. The oxygen-ionic conductivity of LSGM can be further enhanced by the partial substitution of gallium with 3D metals. One of the most investigated dopant cations for LSGM is cobalt. The introduction of even 5–10% of cobalt cations into the gallium sublattice can significantly increase the oxygen-ionic conductivity of LSGM [22,23]. However, the most promising cation dopant for LSGM application in electrochemical cells with symmetrical electrodes is iron. The partial substitution of gallium with iron allows an increase in the conductivity of LSGM and improves the affinity to the electrode materials based on strontium ferrite, the main electrode material for symmetrical fuel cells [24]. In addition, the thermal expansion of LSGM is expected to increase and become more closer to the thermal expansion of the electrode material, which should have a favorable effect on the quality of the electrode/electrolyte interface [25,26]. However, when LSGM is doped with iron, the concentration of electron charge carriers should increase, which, on the one hand, should lead to an increase in the overall conductivity and, on the other hand, to an increase in the leakage current in the fuel cell mode, so the balance in the Ga/Fe ratio is important. At the same time, with the increasing concentration of electron carriers, the area of the electrochemical reactions (oxygen reduction and hydrogen oxidation) should expand from the electrode/electrolyte/gas (or electrode/gas) interface to the electrolyte/gas interface, which, theoretically, should increase the electrochemical activity of the electrodes.

In this paper, we studied how the introduction of iron cations into the gallium sublattice in La_0.8_Sr_0.2_Ga_0.8-x_Fe_x_Mg_0.2_O_3−δ_ (x = 0–0.2) influences the basic characteristics of the oxide (crystal structure, phase composition, thermal expansion, electrical conductivity in oxidizing and reducing atmospheres, bulk, and grain boundary resistance). In addition, studies of the electrochemical activity of Sr_2_Fe_1.5_Mo_0.5_O_6−δ_ electrodes in contact with La_0.8_Sr_0.2_Ga_0.8-x_Fe_x_Mg_0.2_O_3−δ_ electrolyte were performed, and single fuel cell tests with symmetrical Sr_2_Fe_1.5_Mo_0.5_O_6−δ_ electrodes and a La_0.8_Sr_0.2_Ga_0.8-x_Fe_x_Mg_0.2_O_3−δ_ supporting electrolyte were carried out.

## 2. Materials and Methods

### 2.1. Sample Preparation and Characterization

Powders with the composition of La_0.8_Sr_0.2_Ga_0.8-x_Fe_x_Mg_0.2_O_3−δ_ (x = 0–0.2, hereafter LSGF_x_M) were prepared by the conventional solid-state technique. La_2_O_3_ (99.9 wt.%), SrCO_3_ (99.99 wt.%), Ga_2_O_3_ (99.9 wt.%), MgO (99.5 wt.%), and Fe_2_O_3_ (99.5 wt.%) were used as initial reagents. The reagents in the required stoichiometric ratio were ball-milled with zirconium oxide balls in a planetary ball mill, PM 100 (Retsch^®^, Haan, Germany), at 250 rpm for 1 h. The resulting powder was annealed in a muffle furnace at 1000 °C for 6 h in air, followed by regrinding in the planetary ball mill. To obtain dense ceramic samples, the annealed powders were pressed into the form of bars and pellets by uniaxial cold-pressing at a pressure of 3 tons/cm^2^, followed by sintering at a heating and cooling rate of 100 °/h at 1450 °C for 6 h in air. The obtained ceramic samples had a relative density of about 96%. 

The single-phase Sr_2_Fe_1.5_Mo_0.5_O_6−δ_ electrode powder (hereafter SFM) was synthesized by the solid-state method with a final synthesis temperature of 1100 °C for 2 h in air. The detailed methodology of the powder synthesis and its certification is described in [27].

The phase composition of the obtained LSGF_x_M powders was studied by the X-ray diffraction method using CuK_α_ radiation (λ = 1.5418 Å), with steps of 0.01° in the 2θ range 20–90° using a D/MAX-2200 (Rigaku Corporation, Takatsuki, Japan). To determine the chemical composition of the synthesized electrolytes, the inductively coupled plasma atomic emission OPTIMA 4300 DV spectroscopy was applied (PerkinElmer Inc., Waltham, MA, USA). The SEM images were obtained on the cross-section of samples in the direction perpendicular to the plane. The SEM images and energy-dispersive X-ray analysis were performed using a VEGA (Tescan, Brno, Czech Republic) electron microscope with an energy-dispersive X-ray microanalysis system INCA Energy 350 (Oxford Instruments, Abingdon, UK). The thermal expansion (TEC) of the ceramics was studied on an automatic quartz dilatometer based on a Tesatronic TT-80 appliance with a GT-21HP probe (TESA, Renens, Switzerland). The measurements were carried out in air at a heating rate of 2 °/min up to 900 °C. The samples for the TEC testing were rectangular bars with a length of about 17 mm and a cross-section of 3 × 3 mm.

### 2.2. Electrical and Electrochemical Measurements

Electrical conductivity measurements were carried out by the conventional 4-probe DC method using a 2700 multimeter (Keithley Instruments, Solon, OH, USA) in an air and wet (3 vol.% H_2_O) hydrogen atmosphere, as well as depending on the oxygen partial pressure (regulated using a solid oxide oxygen pump). Samples for the measurements were in the form of bars with four probes made from platinum wire and platinum paste.

For the electrochemical studies, samples in the form of pellets with symmetrical electrodes were prepared. To study the bulk and grain boundary resistance of the ceramics, the electrodes were made of platinum paste and sintered at 1000 °C for 1 h. Bulk and grain boundary conductivities were calculated using resistances, obtained during the fitting of the impedance data taking into account the geometry of the samples according to Equation (1), where *l* and *S* are sample thickness and area, respectively.

(1) $$\sigma_{b,gb}=(R_{b,gb}\cdot S)/l$$

For electrode kinetics studies and fuel cell tests, SFM electrodes were prepared on the electrolyte pellets. The electrode slurry, prepared by mixing the organic base with SFM powder, was deposited onto both sides of the electrolyte using the doctor blade printing technique with subsequent sintering at 1050 °C for 2 h with a heating and cooling rate of 85 °/h. 

High-temperature experiments were performed in air and wet (3 vol.% H_2_O) hydrogen. The experimental setup is illustrated in [28]. All the measurements were carried out at atmospheric pressure. The electrochemical performance of the electrodes and SOFCs was studied by means of impedance spectroscopy using an Solartron FRA-1260 and an EI-1287 (Ametek, Hampshire, UK). The cell was connected to the electrochemical interface in the two-electrode four-wire mode, which permitted the exclusion of the impedance of current-supplying cables from the overall impedance. The analysis of impedance spectra was performed by the distribution of relaxation times (DRT) method by means of the program code developed by the authors of [29].

## 3. Results

### 3.1. Samples Characterization

In order to determine the quantitative cation composition of the synthesized samples, atomic emission spectroscopy (AES) was performed. The gross formulas of the obtained compounds were calculated based on the assumption that lanthanum and strontium cations occupy only the A position, while gallium, magnesium, and iron cations occupy only the B position in the ABO_3_ perovskite structure. The content of cations in the analyzed samples and the calculated formulas of the obtained samples are presented in Table 1. In general, the obtained compositions correspond well to the specified ones.

**Figure 1:**
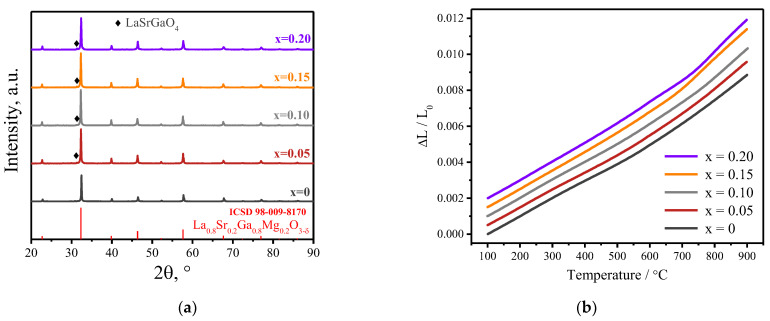
XRD patterns of La_0.8_Sr_0.2_Ga_0.8-x_Fe_x_Mg_0.2_O_3−δ_ (x = 0, 0.05, 0.1, 0.15, 0.2) powders after calcination at 1450 °C in air for 6 h (**a**). Dilatometric curves of the thermal expansion of LSGF_x_M ceramic samples. Dependencies are shifted along the y-axis for better perception (**b**).

The X-ray analysis of the La_0.8_Sr_0.2_Ga_0.8-x_Fe_x_Mg_0.2_O_3−δ_ powders revealed the presence of a small amount of the second phase in all the synthesized samples (Figure 1a), which was identified as LaSrGaO_4_. The formation of this magnesium-free phase has been observed by the majority of researchers [30,31,32], and its formation is associated with lower formation energy compared to the main product. Taking into account its low content (not more than 1 wt.%), we expect that its presence should not affect the main properties of the compounds. 

The dilatometric curves of the LSGF_x_M samples (Figure 1b) show a non-linear behavior similar to that previously obtained for LSGM [33]. According to [34], the transition at about 280 °C is attributed to the change in the crystal structure from orthorhombic to rhombohedral. At the same time, high-temperature neutron diffraction data indicate monoclinic distortions of the LSGM crystal lattice at low temperatures, leading to a series of structural transitions from the pseudo-orthorhombic to the rhombohedral phase [35]. There are also indications of two second-order phase transitions near 500 and 600 °C, accompanied by changes in the oxygen sublattice and the conductivity activation energy [36]. The average TEC values were obtained by linear approximation in two temperature regions and are shown in Table 2. In the low-temperature region of 100–600 °C, a slight increase in TEC is observed with an increase in the iron concentration from 9.8 × 10^−6^ 1/K for LSGM up to 10.8 × 10^−6^ 1/K for LSGF_0.2_M. In the high-temperature range of 650–900 °C, the thermal expansion coefficient increases with the iron concentration from 12.99 × 10^−6^ 1/K for LSGM up to 17.52 × 10^−6^ 1/K for LSGF_0.2_M. In general, with the increasing concentration of iron in LSGF_x_M, TEC increases and becomes comparable to the TEC values for the electrode materials.

### 3.2. Total and Partial Conductivity

**Figure 2:**
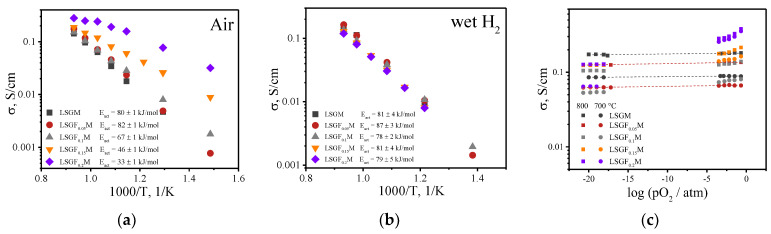
Temperature dependencies of electrical conductivity for LSGF_x_M in air (**a**) and wet hydrogen (**b**). pO_2_ dependencies of electrical conductivity for LSGF_x_M at 700 and 800 °C (**c**).

Electrical conductivity studies have shown that the replacement of 5% of gallium with iron has no effect on either the value of conductivity or the value of activation energy, which was calculated from the slopes of the temperature dependencies of electrical conductivity in an air atmosphere (Figure 2a). As the iron content in the oxide increases, the conductivity of LSGF_x_M increases, and the activation energy value decreases, clearly indicating the appearance of electron/hole charge carriers. The iron cation can exhibit several oxidation degrees (+2/+3/+4). When the trivalent gallium cation is replaced with iron, the introduced iron will mostly have the same oxidation state (+3), but, at high temperatures in an oxidizing atmosphere, some of the iron cations can be oxidized to the +4 oxidation state, according to Equation (2), resulting in the appearance of electronic holes.

(2) $$2Fe_{Ga}^{\times }+V_{O}^{\cdot \cdot }+0.5O_{2}↔O_{O}^{\times }+2Fe_{Ga}^{\cdot }$$

In wet hydrogen, electrical conductivity is almost independent of the concentration of iron in the oxide, as well as the activation energy with the value around 80 kJ/mol, which corresponds to the activation energy value for oxygen-ionic conductivity (Figure 2b). Apparently, in a reducing atmosphere, all the iron cations remain in the +3 oxidation state, which does not lead to the formation of either additional oxygen vacancies or the appearance of electron charge carriers. Studies of electrical conductivity behavior on oxygen partial pressure showed that electrical conductivity for undoped and LSGF_0.05_M samples does not depend on pO_2_ in the whole investigated range (Figure 2c). For the higher iron content in the oxides, electrical conductivity decreases with decreasing pO_2_ in oxidative conditions (pO_2_ > 10^−5^ atm) and does not depend on it in a reductive atmosphere (10^−20^ < pO_2_ < 10^−15^ atm). The decrease in electrical conductivity with the pO_2_ decrease becomes more pronounced with increasing iron content. Such behavior confirms the appearance of an electronic hole contribution to the electrical conductivity at high oxygen pressures and pure ionic transport at low pO_2_.

**Figure 3:**
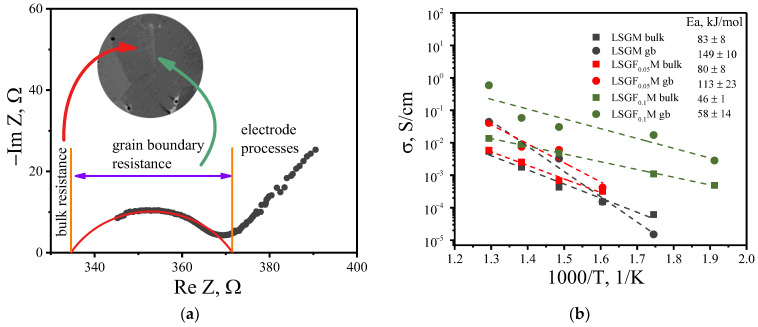
An impedance spectrum for LSGM electrolyte at 400 °C in air (**a**), temperature dependencies for bulk and grain boundary conductivity for investigated oxides (**b**).

To determine the influence of iron doping on the bulk and grain boundary contributions to the total conductivity, impedance studies were carried out. It was found that the separation of the contributions was possible only for undoped LSGM and compositions with 5 and 10% of iron. An example of an impedance spectrum is shown in Figure 3a. The impedance data were analyzed according to an R_b_-(R_gb_-CPE) equivalent circuit, where R_b_ и R_gb_ correspond to the bulk and grain boundary resistances, respectively (resulting fitting parameters are shown in Table 3). From the obtained results, one can see that doping with Fe affects both bulk and grain boundary conductivity, leading to an increase in conductivity values with a decrease in the activation energy values (Figure 3b). 

**Figure 4:**
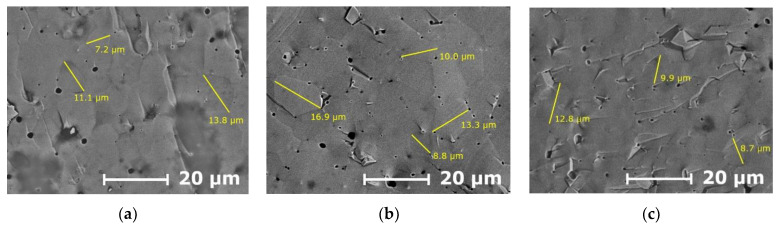
SEM images of the LSGF_x_M ceramics (x = 0 (**a**), x = 0.05 (**b**), x = 0.1 (**c**)).

Figure 4 depict SEM images of the LSGF_x_M (x = 0–0.1) ceramics. One can see that the microstructure almost does not change with Fe doping: the average grain size is about 10 µm. Therefore, all the changes in bulk and grain boundary conductivity come from the introduction of iron into the LSGM. The effect is more pronounced for the grain boundary contribution, which is not surprising since the dopants usually tend to accumulate in the grain boundary region. For higher concentrations of iron, the impedance data did not allow to distinguish bulk and grain boundary contributions. Such behavior suggests that for high Fe concentrations (15 and 20%), the grain boundary conductivity increases to the level where the path of electrical conduction only through grain boundaries is realized. 

### 3.3. High-Temperature Electrochemical Study

**Figure 5:**
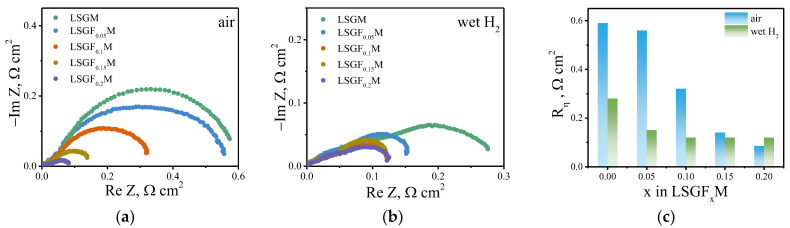
Impedance spectra for Sr_2_Fe_1.5_Mo_0.5_O_6−δ_ electrodes in contact with LSGF_x_M (x = 0–0.2) electrolyte at 800 °C in air (**a**) and wet hydrogen (**b**). Dependencies of polarization resistance of Sr_2_Fe_1.5_Mo_0.5_O_6−δ_ electrode on iron content in the supporting LSGF_x_M electrolyte at 800 °C (**c**).

As was mentioned in the introduction, the partial replacement of gallium with iron in the electrolyte, in theory, should lead to a change in the electrode reaction mechanism and/or an increase in the electrochemical activity of the electrodes due to the expansion of the area of the electrochemical reaction from the electrode/electrolyte/gas (or electrode/gas) interface to the electrolyte/gas interface. To verify this hypothesis, electrochemical cells with symmetrical Sr_2_Fe_1.5_Mo_0.5_O_6−δ_ (SFM) electrodes and a LSGF_x_M (x = 0–0.2) supporting electrolyte were prepared. The studies were performed in both an air and a wet hydrogen atmosphere. Figure 5 shows the impedance spectra of the electrochemical cells in different atmospheres at 800 °C. As can be seen from the data, in the air atmosphere, the polarization resistance of the SFM electrode is mainly determined by the contribution of the low-frequency semicircle (Figure 5a). In the wet hydrogen atmosphere, the impedance spectrum consists of several overlapping semicircles, which are difficult to distinguish (Figure 5b). Such behavior in different atmospheres is caused by different mechanisms of the oxygen reduction and hydrogen oxidation reactions and by the different nature of the rate-determining stages of these electrode reactions. The slow rate of oxygen interphase exchange with the gas phase limits the oxygen reduction reaction [37], while the slow rate of dissociative adsorption of hydrogen on the SFM surface determines the hydrogen oxidation reaction [38]. The most significant information from these data is that as the iron content in the supporting electrolyte increases, the polarization resistance of the SFM electrode decreases in both air and wet hydrogen atmospheres. In the air, the polarization resistance decreases approximately six times in the range of studied iron concentrations (Figure 5c). In a reducing atmosphere, a two-time decrease in the polarization resistance is observed when 5% gallium is replaced with iron in the LSGF_x_M electrolyte. A further increase in the iron content has almost no effect on the polarization resistance of the electrode in a reducing atmosphere (Figure 5c).

**Figure 6:**
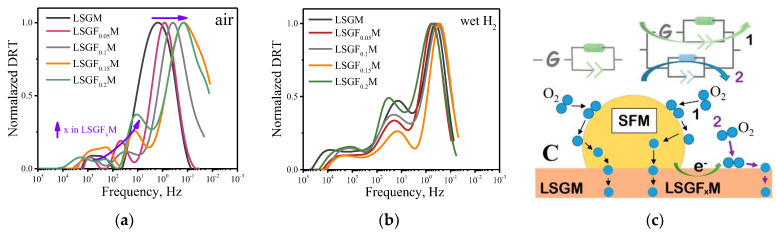
DRT functions for Sr_2_Fe_1.5_Mo_0.5_O_6−δ_ electrode in contact with LSGF_x_M (x = 0–0.2) electrolyte at 800 °C in air (**a**) and wet hydrogen (**b**). Sketch of the oxygen reduction reaction for the cases of LSGM and LSGF_x_M supporting electrolytes (**c**).

In order to understand the reasons for the decrease in the polarization resistance of the electrodes, let us consider the behavior of the DRT functions. Figure 6 shows the normalized DRT functions calculated from the impedance spectra in Figure 5. One can see that, in the air atmosphere, in the case of the LSGM electrolyte without iron, there are two peaks on the DRT function in the frequency region around 500 and 1 Hz. As the iron content in the electrolyte increases, there is a uniform shift of the low-frequency peak to the lower-frequency region. At the highest iron content, the relaxation frequency of the low-frequency peak is about 0.1 Hz. The relaxation frequency of the high-frequency peak also shifts to the low-frequency region, and peak splitting also occurs. This clearly indicates a change in the mechanism of the electrode reaction of oxygen reduction during iron doping of the supporting LSGM electrolyte. On the other hand, in a humid hydrogen atmosphere, the concentration of iron in the electrolyte has almost no effect on the DRT function and the relaxation frequencies of the peaks, suggesting that the mechanism of the hydrogen oxidation reaction does not depend on the iron content in the supporting electrolyte.

As was mentioned above in the discussion of conductivity behavior, in an oxidizing atmosphere, iron cations partially exist in a +4 oxidation state, which leads to the appearance of electronic charge carriers in the electrolyte. Earlier, using the oxygen isotope exchange method, it was shown that the introduction of 3D metal into the gallium position in LSGM leads to an increase in both the rate of oxygen interfacial exchange between the electrolyte and the gas phase, and in the oxygen diffusion coefficient in the electrolyte [39]. This suggests that, in the case of electrochemical cells with the LSGM electrolyte doped with iron, a parallel pathway of the oxygen reduction reaction appears on the surface of the LSGF_x_M electrolyte, schematically shown in Figure 6c. Previously, it was shown that the oxygen reduction rate on the SFM electrode in contact with the LSGM electrolyte can be described by an equivalent electrical circuit consisting of a series-connected Gehrischer element and one ZARC element (see [27] for more details). When electronic charge carriers appear in the electrolyte, active centers for oxygen adsorption and dissociation appear on the electrolyte surface, where oxygen reduction can take place. The electrons in this case are supplied from the SFM electrode along the electrolyte surface to the reaction zone and the oxygen reduction can be described by two parallel reaction pathways, one of which is similar to the one for LSGM (designated as 1 in Figure 6c). The second pathway can include the stages of electron transport in the electrolyte and oxygen adsorption and dissociation on the LSGF_x_M surface (designated as 2 in Figure 6c). It is obvious that as the iron content increases, the rate of the second pathway will increase, due to an increase in the concentration of electronic charge carriers. 

### 3.4. SOFCs Testing

**Figure 7:**
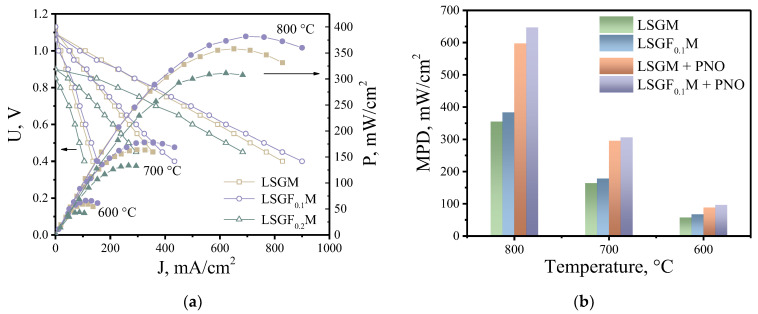
I-V and I-P curves for investigated fuel cells (**a**). Comparison of the maximum power density (MPD) for fuel cells with initial electrodes and after their impregnation with a saturated solution of Pr_2_NiO_4+δ_ (**b**).

Studies of electrochemical cells in a fuel cell mode were performed on samples with a LSGF_x_M (x = 0, 0.1, 0.2) supporting electrolyte and with symmetrical SFM electrodes. The thickness of the supporting electrolyte was 0.55 mm in all cases. Figure 7a shows the current-voltage (I-V) and current-power (I-P) curves of the investigated fuel cells when atmospheric air was supplied to the cathode and wet hydrogen to the anode. The cell with the LSGF_0.1_M supporting electrolyte showed the best characteristics. The cell power density reached values of about 380, 180, and 80 mW/cm^2^ at 800, 700, and 600 °C, respectively, which is a good result for the fuel cell with the supporting electrolyte [16,24]. The fuel cell with the LSGM electrolyte showed slightly inferior performance due to the lower conductivity of the electrolyte without the iron addition, and the lower electrochemical activity of the electrodes in contact with LSGM electrolyte compared to LSGF_0.1_M. The LSGF_0.2_M-supported fuel cell showed much worse performance due to the low OCV caused by the significant contribution of electronic conductivity in LSGF_0.2_M. It is known that the electrochemical activity of SFM electrodes, especially in an oxidizing atmosphere, does not exhibit high values [24], which makes it difficult to evaluate the influence of the supporting electrolyte on the performance characteristics of the cell due to the significant contribution of electrode polarization. To improve the power characteristics of the fuel cell by reducing the polarization resistance, the electrodes were impregnated with a saturated solution of Pr_2_NiO_4+δ_, which has previously been shown to be an effective electrocatalyst for symmetrical electrodes [40]. After impregnation, the power of the fuel cell with the LSGF_0.1_M supporting electrolyte increased to about 650 mW/cm^2^ at 800 °C, Figure 7b. The cell with the undoped electrolyte showed a slightly worse result, about 600 mW/cm^2^ at 800 °C. As can be seen in Figure 7b, the difference between the power density of the cells in the original state and with the impregnated electrodes becomes insignificant as the temperature decreases. This is due to the larger contribution of the cell ohmic resistance compared to the polarization of the electrodes in the overall voltage drop with decreasing temperature. To improve the efficiency of such cells at lower temperatures, it is necessary to increase the specific conductivity of the supporting electrolyte and reduce its thickness, which will be the focus of further studies. 

## 4. Conclusions

In this study, the results of the investigation of a highly conductive solid electrolyte, based on (La,Sr)(Ga,Mg)O_3_ with the partial substitution of gallium with iron cations, have been presented. It was found that the coefficient of thermal expansion increases with increasing iron content, which has a positive effect in terms of thermo-mechanical compatibility between the electrode and electrolyte materials. Conductivity studies have shown that in an air atmosphere with increasing iron content, the conductivity increases and the activation energy decreases, which is explained by the appearance of electronic conductivity in the material. In a humid hydrogen atmosphere, the conductivity and the activation energy are almost independent of the iron content, probably due to the fact that the iron cations remain in the +3 oxidation state in a reducing environment. It is shown that the electrochemical activity of Sr_2_Fe_1.5_Mo_0.5_O_6−δ_ electrodes in contact with a (La,Sr)(Ga,Fe,Mg)O_3_ electrolyte increases with increasing iron content in the electrolyte in an air atmosphere, which is explained by the appearance of a parallel oxygen reduction reaction pathway at the air/electrolyte interface. Studies of fuel cells with a 0.55 mm supporting electrolyte showed that cells with a La_0.8_Sr_0.2_Ga_0.7_Fe_0.1_Mg_0.2_O_3−δ_ electrolyte demonstrated the best characteristics. When highly active (impregnated) symmetrical electrodes were used, the maximum power density was about 650 mW/cm^2^ at 800 °C.

## Figures and Tables

**Table 1:** Composition of La_0.8_Sr_0.2_Ga_0.8–x_Fe_x_Mg_0.2_O_3–δ_ samples determined by AES method.

Given Composition	Concentration of the Cations in the Analyzed Sample/wt.%					Composition Calculated Based on the AES Analysis
	La	Sr	Ga	Mg	Fe	
La_0.8_Sr_0.2_Ga_0.8_Mg_0.2_O_3−δ_	46.96	8.14	23.66	1.82	0.01	La_0.78_Sr_0.22_Ga_0.82_Mg_0.18_O_3−δ_
La_0.8_Sr_0.2_Ga_0.75_Fe_0.05_Mg_0.2_O_3−δ_	46.88	8.13	22.60	2.00	1.20	La_0.78_Sr_0.22_Ga_0.76_Fe_0.05_Mg_0.19_O_3−δ_
La_0.8_Sr_0.2_Ga_0.7_Fe_0.1_Mg_0.2_O_3−δ_	48.41	7.74	20.37	1.92	2.41	La_0.8_Sr_0.2_Ga_0.71_Fe_0.1_Mg_0.19_O_3−δ_
La_0.8_Sr_0.2_Ga_0.65_Fe_0.15_Mg_0.2_O_3−δ_	47.38	8.02	19.77	1.96	3.56	La_0.79_Sr_0.21_Ga_0.66_Fe_0.15_Mg_0.19_O_3−δ_
La_0.8_Sr_0.2_Ga_0.6_Fe_0.2_Mg_0.2_O_3−δ_	48.42	7.98	17.75	1.94	4.66	La_0.79_Sr_0.21_Ga_0.61_Fe_0.2_Mg_0.19_O_3−δ_

**Table 2:** TEC values (10^−6^ 1/K) for LSGF_x_M samples.

Sample	100–600 °C	650–900 °C
LSGM	9.75(3)	12.99(6)
LSGF_0.05_M	9.79(3)	13.71(9)
LSGF_0.1_M	10.09(3)	13.90(12)
LSGF_0.15_M	10.63(4)	16.44(8)
LSGF_0.2_M	10.76(4)	17.52(8)

**Table 3:** Fitting parameters for impedance data for LSGF_x_M (x = 0–0.10) at 400 °C.

Sample	R_bulk_, Ω cm	R_gb_, Ω cm	C_gb_, F/cm	f_max_, Hz
LSGM	2340	340	2.6 × 10^−9^	1.1 × 10^5^
LSGF_0.05_M	1469	166	4 × 10^−9^	2.3 × 10^5^
LSGF_0.1_M	206	32.6	1 × 10^−8^	5 × 10^5^

## Data Availability

Not applicable.
